# *Campylobacter* presence on Dutch broiler farms and associated risk factors

**DOI:** 10.1016/j.psj.2024.103568

**Published:** 2024-02-19

**Authors:** Ewa Pacholewicz, Anita Dame-Korevaar, Marleen van der Most, Hilko Ellen, Martien H. Bokma, Miriam G.J. Koene

**Affiliations:** ⁎ Department of Epidemiology, Bioinformatics and Animal models, Wageningen Bioveterinary Research, 8221 RA Lelystad, The Netherlands; † Department of Bacteriology, Host Pathogen Interaction & Diagnostics Development, Wageningen Bioveterinary Research, 8221 RA Lelystad, The Netherlands; ‡ Wageningen Livestock Research, 6708, Wageningen, The Netherlands

**Keywords:** campylobacter, broiler, longitudinal, monitoring, risk factor

## Abstract

*Campylobacter* is the most reported zoonotic pathogen in humans in the European Union. Poultry is a major source of human infection with *Campylobacter.* Although many studies are done on the presence of *Campylobacter* in broilers and theoretically effective control measures are known, their relative importance at broiler farms remains poorly understood. Therefore, the aim of this study was to investigate the presence of *Campylobacter* on selected broiler farms in the Netherlands, to determine the moment of introduction, and associated risk factors. A longitudinal study on 25 broiler farms was carried out between June 2017 and December 2020. Fecal samples were collected weekly from 43 broiler houses. In total 497 flocks were sampled. Putative variables on flock and farm characteristics for a risk factor analysis were gathered through questionnaires. Risk factors associated with the presence of *Campylobacter* in a broiler flock were determined using regression models. In total 30% of the flocks included in the study were positive for *Campylobacter*. Factors associated with presence of *Campylobacter* at slaughter age included: season, mowing lawns and presence of agricultural side activities. While summer/autumn and mowing lawns were associated with an increase in *Campylobacter* presence in flocks, the farmer having agricultural side activities other than poultry production was associated with a decrease. Analysis of the age at which flocks first tested *Campylobacter* positive revealed that slower growing breeds became positive on average 1 wk later compared to regular growers. This study revealed a delayed introduction of *Campylobacter* in slower grower vs. regular grower broiler flocks reared indoors. In addition, it confirmed importance of season as major risk factor. The relevance of mowing and preceding positive flocks as risk factors needs further investigation.

## INTRODUCTION

*Campylobacter* is the most reported gastrointestinal bacterial pathogen in humans in the European Union, as reported by several European and Dutch monitoring studies, which even underestimate the actual numbers (Havelaar et al., 2013; Teunis et al., 2013; Pijnacker et al., 2019; EFSA/ECDC. 2021; Mughini-Gras et al., 2021). Several European risk assessment studies pointed to the importance of broilers as a main source of human campylobacteriosis (EFSA. 2010; EFSA. 2011; Mughini Gras et al., 2012; Mughini-Gras et al., 2021). At this moment the surveillance of *Campylobacter* in broilers in the Netherlands is based on sampling random flocks at slaughterhouse level. Since January 2018 the Process Hygiene Criterion (PHC) of 1,000 CFU/g of *Campylobacter* on the neck skins of chilled broiler carcasses is enforced (Commission Regulation (**EU**) 2017/1495 of 23 August 2017 amending Regulation (**EC**) No 2073/2005). Upon unacceptable PHC results, the slaughterhouses are responsible to improve the processing hygiene. In the Netherlands in 2020 there were 34 % positive flocks reported with 9.3% neck skin samples showing levels above 1,000 CFU/g (Anonymous 2022).

The theoretically most effective *Campylobacter* control measures on farms were recently ranked by experts providing the order of relative risk reduction of selected measures as follows: vaccination, feed and water additives, discontinued thinning, employing a limited number and well-trained staff, avoiding drinkers that allow standing water, addition of disinfectants to drinking water, hygienic anterooms, and designated tools per broiler house (EFSA. 2020). Unfortunately, many of the measures are difficult to implement (e.g., discontinued thinning, employing a limited number and well-trained staff, avoiding drinkers that allow standing water; hygienic anterooms, and designated tools per broiler house), have insufficient effect in practice (e.g., feed and water additives, addition of disinfectants to drinking water) or are not yet available (e.g., vaccination). Thus, farmers have limited solutions in hands to prevent *Campylobacter* introduction in flocks, except from complying to biosecurity measures at the best level possible. Also, it is known that after introduction in a poultry flock *Campylobacter* spreads readily within the flock, resulting in >90% of all birds excreting *Campylobacter* a few days after introduction until the end of the production cycle (Wagenaar et al., 2013). Therefore, more detailed information on the time and risk factors regarding the introduction and transmission of *Campylobacter* in broiler flocks is needed to properly advise farmers.

In 2015, a project started in the Netherlands, financially supported by the Dutch Ministry of Agriculture, Nature and Food Quality), the primary broiler production sector (AVINED), and the Association of Dutch Poultry Processing Industries (NEPLUVI), together with research institutions (Wageningen Bioveterinary Research, Wageningen Livestock Research, Veterinary Faculty of Utrecht University). The overall aim of this project was to investigate ways to reduce *Campylobacter* both at farm level and in the slaughterhouse. One of the projects’ research goals, described under this study, was to investigate *Campylobacter* presence on Dutch broiler farms, the moment of introduction, and associated risk factors.

## MATERIAL AND METHODS

### Study Design

*Investigated farms.* Inclusion of broiler farms was done on a voluntary basis and the farmers were approached through the network of researchers and a large veterinary poultry practice. Twenty-five broiler farmers agreed to participate in the study, 16 participated during the entire study period and the others stopped or joined half way. A longitudinal study was carried out from June 2017 until December 2020. In 2017-2018 there were 1 to 3 broiler houses included per farm location, whereas from 2019 onwards only one randomly selected house per farm was included. In total, 43 houses and 497 flocks were sampled, all reared indoors, where a flock is defined as a group of chickens raised together in one house during one rearing cycle.

### Sample Collection

Farmers were asked to collect pooled fecal samples weekly from each house included in the study, starting in general from the second week after arrival of the chickens on the farm (after hatching) until slaughter of the flock. Fecal samples were collected in a plastic container, by walking through the house and picking up fresh fecal material from 4 to 6 different locations in the house. The fecal samples were labelled and stored by the farmers in a -20°C freezer. At the end of the rearing cycle the set of samples was collected by a member of the research team and delivered to the National Reference Laboratory for *Campylobacter* at WBVR in Lelystad for PCR testing.

### Explanatory Variables Data Collection

Farmers were asked to provide information about the characteristics of their farm and flocks through a questionnaire. (Supplementary Material Table 1 and 2). Moreover, to gather information about the flock characteristics, the farmers were asked to fill in log books about activities that took place in and around the broiler house during the rearing cycle (Supplemental Material Table 3). From 2019 this logbook was replaced by a selection of specific questions on the sample submission forms (Supplemental Material Table 4). In addition, for each flock the farmers provided information through the Food Chain Information form (VKI Voedselketen informatie, Supplemental Material Table 5), which is an obligatory form requested by Dutch slaughterhouses prior to slaughter. Based on the questionnaire, the logbook or submission form and the Food Chain Information form, 35 putative risk factors were determined and included in the analysis, as described under the section “Statistical analysis” and presented in Table 1.

**Table 1:** Overview of the explanatory variables gathered during the monitoring study on 25 participating farms. The variables were gathered on a flock, farm or broiler house level. The total number of flocks was 497.

Explanatory variables (flock level)		Levels	*n* flocks (max 497)
Breed		Cobb	9
		JA57 (Hubbard 257, 757)	99
		JA87 (Hubbard 287, 787, 987)	88
		Ranger (Gold, Ranger)	84
		Ross 308	152
		not reported	65
Production concept	Regular and slower growers	Regular growers (Cobb, Ross 308)	161
		Slower growers (JA, Ranger)	271
		not reported	65
Number of animals	Number of animals in a flock	<15,000	99
		15,000 – 25,000	87
		25,000 – 40,000	186
		>40 000	58
		not reported	67
Diseases	Registered presence of diseases during rearing cycle	No	335
		Yes	89
		not reported	73
Antibiotics	Registered use of antibiotics during rearing cycle	No	143
		Yes	80
		not reported	274
Thinning	Partial depopulation of the flock	No	261
		Yes	160
		not reported	76
*Salmonella* status	*Salmonella* status of the flock	Negative	374
		Positive	29
		not reported	94
Mowing lawns	Reported mowing activities around the broiler house during a rearing cycle	No	179
		Yes	190
		not reported	128
Agricultural activities	Agricultural activities observed in the vicinity of the farm performed during rearing cycle (for example mowing lawns, ploughing, fertilizing land or fields)	No	253
		Yes	117
		not reported	127
Maintenance	Maintenance inside the broiler house during rearing cycle	No	288
		Yes	69
		not reported	140
Visitor house	Visitor in a chicken house without wearing protective clothes	No	336
		Yes	33
		not reported	128
Hatchery	Hatchery (anonymized)	A	101
		B	56
		C	23
		D	113
		E	67
		Others (pooled 9 hatcheries providing <4% of flocks)	58
		not reported	79
Feed supplier	Feed supplier (anonymized)	A	23
		B	57
		C	155
		D	34
		E	85
		Others (pooled 7 suppliers providing <4% of flocks)	59
		not reported	84
Slaughter age	Age [days] when the flock was slaughtered (categories)	< 40	58
		40 – 50	282
		> 50	140
		not reported	17
Preceding positive flock	*Campylobacter* presence in preceding flock	Negative	278
		Positive	111
		not reported	108
Downtime	Number of days between 2 rearing cycles	<=7	200
		> 7	146
		not reported	151
Season	June-November	Summer/Autumn	298
	December-May	Winter/Spring	199
Stocking density	Number of birds/m^2^	<= 17	274
		18 - 21	45
		> 21	51
		not reported	127
Mortality	Daily mortality at final depopulation [%]	Min	0,7
		Mean	2,7
		Max	10,9
		not reported	394
Explanatory variables (farm level)		Levels	*n* farms (max 25)
Side activity any	Any work related side activity next to broiler farming	No	6
		Yes	11
		not reported	8
Side activities animals	Side activity involving animals (other than broilers)	No	14
		Yes	3
		not reported	8
Agricultural side activities	Agricultural activities next to broiler farming	No	8
		Yes	9
		not reported	8
Presence of animals other than broilers on a farm	Presence of animals other than broilers on a farm, as listed in the questionnaire	No	1
		Yes	16
		not reported	8
Animals running free	Animals (other than broilers) that are expected to roam freely on the farm	No	1
		Yes	16
		not reported	8
Animals confined	Animals (other than broilers) that are expected to NOT roam freely on the farm	No	9
		Yes	8
		not reported	8
Cattle	Presence of cattle on the farm	No	16
		Yes	1
		not reported	8
Horse	Presence of horses on the farm	No	15
		Yes	2
		not reported	8
Sheep	Presence of sheep on the farm	No	14
		Yes	3
		not reported	8
Cat	Presence of cats on the farm	No	12
		Yes	5
		not reported	8
Dog	Presence of dogs on the farm	No	2
		Yes	15
		not reported	8
	Presence of animal species other than specific species included in the questionnaire	No	13
Animal other		Yes	4
		not reported	8
Number of houses	Number of broiler houses per farm (category)	1-4	14
		≥4	8
		not reported	3
Proximity of other poultry farm <2 km		No	6
		Yes	11
		not reported	8
Explanatory variables (broiler house level)		Levels	*n* broiler houses (max 43)
Type drinkers		Nipples with cups	24
		Nipples without cups	5
		not reported	14
House building age	Age of a broiler house [years]	Min	6
		Mean	21
		Max	43

### Farm and Flock Characteristics

The overview of the 35 explanatory variables, grouped on farm and flock level, are displayed in Table 1. It total 497 flocks were sampled from 25 participating farms. Not for all 497 flocks all information was provided; the number of missing observations on flock level is indicated in Table 1.

Out of 25 investigated farms, ten of the farms had a production system using regular growers and ten used slower growing breeds. Five of the farms had produced both types of breeds within the time frame of the study. Of the included flocks, 37% (161/432) were regular growing flocks (i.e. conventional broiler flocks), with Ross 308 being the main breed (152/161). Further, 63% (271/432) were slower growing flocks, produced by parental flock lines JA57, JA87 and Ranger. Flocks originated from 15 hatcheries, of which 2 (A, D) delivered 51% (214/418) of the sampled flocks. The average slaughter age during the study was 48 d, with 41 d for regular breeds and 52 d for the slower growing breeds. Thinning was performed for 85% of the regular growing flocks (135/158). Also, a limited number of slower growing flocks (7/228) were partially depopulated during the rearing. For 18 flocks that were thinned no information on flocks’ breed was available. Feed was delivered by 12 different suppliers, of which 2 (C, E) provided feed to more than half of the flocks (240/413, 58%).

### Laboratory Analytical Methods

Fecal samples were analyzed at the National Reference Laboratory for *Campylobacter* at WBVR using a real-time PCR test (Josefsen et al., 2004) that has been in-house validated for detection of *Campylobacter* in fecal samples from poultry. The results were reported as negative (no signal or Ct values above 40), positive (sigmoid curve and Ct-value ≤36), or inconclusive (dubious). An inconclusive PCR result (Ct-values between 36 and 40) can either point to low numbers of *Campylobacter* in the sample (<100 cfu/gram) or be the result of nonspecific reactions. For the analysis in this study, dubious samples were considered as negative samples, unless a dubious sample was preceded and/or followed by a positive sample. The rationale for this is that after colonization with *Campylobacter* broiler flocks will continue to excrete *Campylobacter* until slaughter (Newell and Fearnley, 2003). Flocks were considered as *Campylobacter* positive when at least one fecal sample collected during the rearing cycle of that specific flock tested positive. Due to budget restrictions, for some flocks sampled in 2019, only the samples from the week before slaughter were analyzed. In case of a positive or dubious result, all samples from the entire rearing cycle were analyzed to determine the time (i.e. sampling date) of the first positive sample.

### Statistical Analysis

The data collected by the questionnaires, logbook or sample submission form and VKI was converted into 35 putative variable risk factors, based on biological relevance, that were included in the data analysis (Table 1). The variables were grouped in flock and farm related variables. Risk factor analysis was done on 2 models. The first model addressed factors associated with presence or absence of *Campylobacter* in a flock at slaughter age (model 1). The second model aimed at identifying risk factors associated with the age of broilers when testing *Campylobacter* positive for the first time and used only data from positive flocks (model 2). The first day of testing *Campylobacter* positive was used to determine the age in days at the moment of introduction of *Campylobacter* in the flock (i.e. date of first positive sample minus the date of arrival in the broiler house). Both models followed the same steps (the first step to build model 1 and 2 was a univariable analysis followed by multivariable analysis) and were conducted at flock level. In both models, the explanatory variables were modelled as fixed effects, with the *farm* as a random effect. This accounted for variation between the farms, since the flocks were clustered in farms. Explanatory variables were selected for the multivariable analysis if they had a *p*-value below 0.25 (Wald *p*-value) in the univariable analysis and less than 30% missing observations, leading to inclusion of variables having entries from at least 70% of the flocks. In addition, variables that were highly associated with the variable *production concept,* that is either regular or slower growers (Chi-square test with *p*-value <0.001, definition of the different concepts in Table 1), were excluded from the multivariable analysis, and instead the variable *production concept* was included (Table 2 and Table 4). Further variable reduction was based on biological relevance, based on scientific knowledge about *Campylobacter*. Associations between remaining variables were checked using the Chi-square test or Fisher exact tests. Variables with *p*-value <0.001 were considered as associated and we avoided including them together in the multivariable analysis. Best fitting multivariable models were obtained by backward selection, choosing the model with the lowest Akaike Information Criterion (AIC) value and including only the flocks without missing observations for the selected variables. The first model (glmer) and the second model (glm), were performed in R software, package lme4 (Bates et al., 2015, R Core Team 2022).

**Table 2:** Results from univariable analysis of *Campylobacter* presence at slaughter age, per explanatory variable, including the Odds Ratio (OR) and 95% Confidence Interval (CI), the total number of observations, the number of *Campylobacter* negative vs. positive observations and missing (number and %) observations. Associations with production concept is based on Chi-squared test. The total number of flocks was 497.

Explanatory variable	OR	95% CI	number of observations	*Campylobacter* negative observations	*Campylobacter* positive observations	*Campylobacter* positive flocks [%]	number of missing observations	% missing observations	Association with production concept (*p* value)
Breed							65	13%	<0.001
Ross 308	reference		152	100	52	34.21			
Cobb	11.06	2.00-94.39	9	2	7	77.78			
JA57	0.72	0.26-1.94	99	66	33	33.33			
JA87	0.45	0.16-1.14	88	66	22	25.00			
Ranger	0.35	0.12-0.95	84	67	17	20.24			
Thinning							76	15%	<0.001
No	reference		261	191	70	26.82			
Yes	1.83	0.95-3.57	160	101	59	36.88			
Mowing lawns*							128	26%	0.423
No	reference		179	138	41	22.91			
Yes	2.12	1.29-3.50	190	115	74	38.95			
Agricultural activities							127	26%	0.947
No	reference		253	184	69	27.27			
Yes	1.94	1.15-3.30	117	69	48	41.03			
Maintenance*							140	28%	0.564
No	reference		288	203	85	29.51			
Yes	1.71	0.9-3.26	69	40	29	42.03			
Visitor house*							128	26%	0.191
No	reference		336	225	111	33.04			
Yes	0.22	0.06-0.63	33	29	4	12.12			
Agricultural side activities*							49	10%	1.000
No	reference		164	94	70	42.68			
Yes	0.38	0.17-0.87	284	223	61	21.48			
Animal other							49	10%	0.094
No	reference		318	209	109	34.28			
Yes	0.34	0.11-0.92	130	108	22	16.92			
Side activity any							49	10%	0.753
No	reference		139	82	57	41.01			
Yes	0.47	0.18-1.19	309	235	74	23.95			
Production concept*							65	13%	
Slow growers	reference		271	199	72	26.57			
Regular growers	2.20	1.07-4.78	161	102	59	36.65			
Hatchery							79	16%	<0.001
A	reference		101	65	36	35.64			
B	0.09	0.02-0.49	56	53	3	5.36			
C	0.24	0.04-1.45	23	20	3	13.04			
D	0.82	0.29-2.32	113	74	39	34.51			
E	1.03	0.37-2.89	67	46	21	31.34			
Others	0.94	0.33-2.69	58	37	21	36.21			
Preceding positive flock*							108	22%	0.049
Negative	reference		278	220	58	20.86			
Positive	2.21	1.29-3.76	111	61	50	45.05			
Season*							0	0%	0.105
Winter/Spring	reference		199	174	25	12.56			
Summer/Autumn	5.67	3.46-9.66	298	173	125	41.95			
House building age							49	10%	<0.001
	0.98	0.95-1.01							
Stocking density							127	26%	<0.001
<=17	reference		274	203	71	25.91			
18-21	2.74	1.00-7.88	45	28	17	37.78			
>21	2.84	1.07-7.87	51	30	21	41.18			
Number of animals							67	13%	<0.001
<= 15 000	reference		99	69	30	30.30			
15 000 - 25 000	1.17	0.42-3.30	87	60	27	31.03			
25 000 - 40 000	1.26	0.47-3.64	186	134	52	27.96			
>= 40 000	2.08	0.62-7.56	58	38	20	34.48			

⁎ Included in the multivariable analysis model 1.

## RESULTS

### Campylobacter Presence in Flocks at Slaughter Age

**Figure 1:**
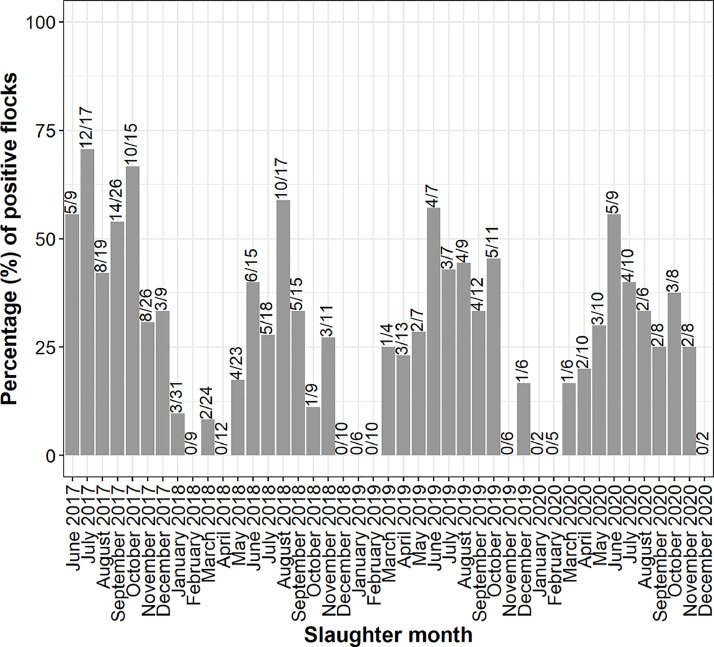
Percentage and number of flocks becoming positive for *Campylobacter* for each study month June 2017 until December 2020. Number of positive flocks and number of flocks tested is indicated above each bar and separated with a slash (number positive/number tested locks).

The monthly percentage of positive flocks ranged from zero to 71%, showing a clear seasonal trend. Overall 30.2% (150/497) of the flocks tested in the period June 2017 until December 2020 were positive for *Campylobacter.* This was varying from 49% in 2017, 20% in 2018, 28% in 2018 and 29% in 2020 (Figure 1).

### Risk Factors Associated With the Presence of Campylobacter in Broiler Flocks (Model 1)

Results from the univariate analysis of *Campylobacter* presence at slaughter age reveled variables (with *p*-values below 0.25, Table 2) to be included in in the second step of building model 1, that is, multivariable analysis. Variables as *breed, thinning, hatchery* and *flock density*, were excluded from multivariable analysis due to their association with *production concept* (*p* <0.001, Chi-squared test). Further, the variable *agricultural activities* (performed during rearing cycle) was excluded due to association with *mowing lawns* (*p* < 0.001, Chi-squared test). The variable *animal other* was excluded since only 4 farms reported having those. In addition, this variable was found to be associated with *agricultural side activities* (*p =* 0.0017). The remaining seven variables which were selected for the multivariable analysis included *mowing lawns, maintenance, visitor in house* without wearing protective clothes, *agricultural side activities, production concept, preceding positive flock* and *season*. After backwards elimination of the variables (from one with the highest *p*-value, to the lowest, and AIC comparison), the results revealed 3 risk factors associated with the *Campylobacter* presence in flocks, as presented in the Table 3, leading to the best fit of model 1, including the results of 292 flocks. Summer/autumn was associated with nearly six times higher risk for the presence of *Campylobacter* in a flock (OR = 5.59, 95% confidence interval (CI) 2.43 – 14.15). The variable *agricultural side activities* was identified as a protective factor, decreasing the odds of a flock testing positive for *Campylobacter* (OR = 0.34, 95% CI 0.11 – 1.03). *Mowing lawns* was kept in the model as it was a confounder factor to season and indicated, although not statistically significant, increased odds for positive flocks by nearly 2 times (OR = 1.74, 95% CI 0.82–3.65).

**Table 3:** Results of multivariable analysis of *Campylobacter* presence at slaughter age (model 1), including the Odds Ratio (OR), 95% Confidence Interval (CI) and *p*-value, the total number of observations and the number of *Campylobacter* negative vs. positive observations.

Explanatory variable		OR	95% CI	*P* value	number of observations	*Campylobacter* negative observations	*Campylobacter* positive observations
Season							
	Winter/Spring (ref)				91	10	81
	Summer/Autumn	5.59	2.43-14.15	<0.001	154	65	89
Mowing lawns							
	No (ref)				123	23	100
	Yes	1.74	0.82-3.65	0.141	122	52	70
Agricultural side activities							
	No (ref)				94	43	51
	Yes	0.34	0.11-1.03	0.044	151	32	119

### Day of First Detection of a Campylobacter Positive Flock and Risk Factors (Model 2)

**Figure 2:**
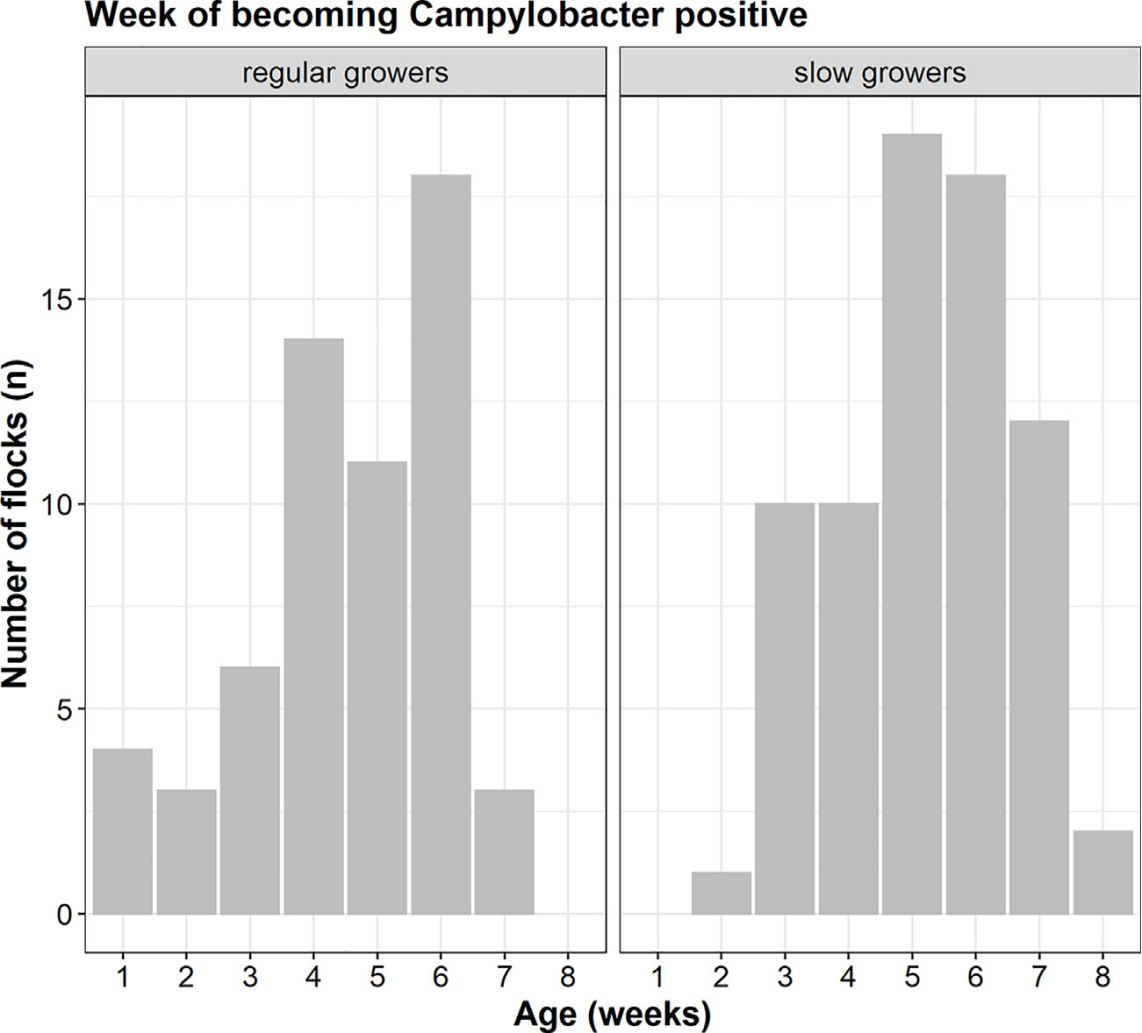
Frequency diagram of the first positive *Campylobacter* test result (age in weeks). The figures summarize results of 144 out of 150 positive flocks in the study (for 6 flocks relevant information to estimate age of first positive sample was missing). Flocks were reared in different concepts (59 positive regular flocks, 72 positive slower grower flocks, for 19 flocks the production concept was unknown), thus slaughtered at different ages. The number of positive flocks decreased after wk 6, since regular growers are slaughtered at that age, thus not sampled anymore.

The mean age of a flock when first testing positive for *Campylobacter* was at 32.60 d (varying from 4 to 56 d). Most flocks had their first positive test result in the 5th or 6th wk of the rearing cycle (Figure 2). To find the risk factors associated with the moment of introduction of *Campylobacter* in a broiler flock, a similar approach as described for model 1 was used. However, in model 2 only data from flocks that were shown to be positive for *Campylobacter* were included (*n* = 150). In step 1, the univariable analysis, the following variables were found to be significantly (*p* < 0.05) associated with the first day of testing *Campylobacter* positive: *production concept* (for regular growers the estimated age of testing positive is 29.62 d, vs. 35.06 for slower growers), presence of *sheep* (33.62 d if no sheep are present, 26.58 if sheep are present), *slaughter age* (23.97 d for flocks slaughtered at an age <40 d, 33.14 for slaughter age 40-50 d and 35.24 for slaughter age >50 d) and *stocking density* (34.91 d for density <17 broilers/m^2^, 31.42 for density 18 to 21, 24.72 for density >21). Details on selected variables based on the univariable analysis are shown in Table 4.

**Table 4:** Results of the univariable analysis of day of testing *Campylobacter* positive, per explanatory variable, including the estimate in days, standard error (SE) and *p*-value, the total number of observations and missing (number and %) observations. Associations with production concept is based on Chi-squared test.

Explanatory variable		Estimate (days)	SE	*P* value	number of observations	number of missing observations	% missing observations	Association with variable production concept
Production concept*						19	13	
	Regular growers (ref)	29.62	1.54		59			
	Slower growers	5.44	2.00	0.009	72			
Breed (categories)						19	13	<0.001
	Ross 308 (ref)	29.43	1.58		52			
	Cobb	1.11	4.18	0.791	7			
	JA57	7.17	2.55	0.009	33			
	JA87	3.88	2.77	0.168	22			
	Ranger	4.97	3.00	0.103	17			
No of animals (categories)						21	14	<0.001
	<15000 (ref)	34.98	2.59		30			
	>40000	0.45	3.78	0.907	20			
	15000-25000	−3.42	3.14	0.279	27			
	25000-40000	−4.28	3.18	0.189	52			
Antibiotics						88	59	0.002
	No (ref)	33.47	1.70		37			
	Yes	−4.09	2.62	0.128	25			
Thinning						21	14	<0.001
	No (ref)	34.22	1.43		70			
	Yes	−3.51	2.02	0.090	59			
Agricultural activities (performed during rearing cycle)*						33	22	0.105
	No (ref)	33.70	1.61		69			
	Yes	−2.51	2.07	0.227	48			
Sheep						19	13	<0.001
	No (ref)	33.62	0.97		115			
	Yes	−7.04	2.71	0.019	16			
Animal other*						19	13	0.041
	No (ref)	33.51	1.18		109			
	Yes	−3.73	2.76	0.192	22			
Presence of animals other than broilers on farm						19	13	0.088
	No (ref)	38.00	4.45		7			
	Yes	−5.48	4.59	0.247	124			
Hatchery*						27	18	0.103
	A (ref)	31.92	2.24		36			
	B	−4.21	6.52	0.521	3			
	C	7.57	6.52	0.250	3			
	D	−0.01	3.20	0.996	39			
	E	4.12	3.56	0.256	21			
	Others	0.69	3.49	0.845	21			
Feed supplier						28	19	<0.001
	A (ref)	27.47	4.90		6			
	B	5.36	5.94	0.376	20			
	C	6.85	5.29	0.205	46			
	D	5.61	6.18	0.372	12			
	E	7.40	5.72	0.203	15			
	Others	1.01	5.74	0.861	23			
Slaughter age (categories)						7	5	<0.001
	<40 (ref)	23.97	2.38		18			
	40-50	9.17	2.57	0.001	80			
	>50	11.27	2.84	<0.001	45			
Preceding positive flock*						42	28	0.525
	No (ref)	33.51	1.50		58			
	Yes	−2.99	1.86	0.110	50			
Number of houses (categories)*						8	5	1
	1-3 (ref)	30.87	1.39		92			
	4	4.37	2.31	0.078	50			
Stocking density (categories)						41	27	<0.001
	<17 (ref)	34.91	1.24		71			
	18-21	−3.49	2.60	0.183	17			
	>21	−10.16	2.43	<0.001	21			

⁎ Included in the multivariable analysis model 2.

For the second step of building model 2, the multivariable analysis, the following variables were selected based on the same selection criteria as for model 1: *production concept, agricultural activities* (performed during rearing cycle), *other animals* (than broilers) present at the farm, *hatchery, preceding positive flock, number of houses*. After backwards elimination of the variables, the risk factors as presented in the Table 5 led to the best fit of model 2, based on the results of 71 flocks. The results of this final model show that slower growers were found to be *Campylobacter* positive later during the rearing cycle compared to regular growers (+6.50 d, 95% CI 1.25–11.60). All other variables in the model had no significant influence on the estimated first day of testing *Campylobacter* positive. Backward elimination of these variables did not improve the model fit, thus they remained in the model.

**Table 5:** Results of the multivariable analysis of day of testing *Campylobacter* positive (model 2), including the estimate in days, standard error (SE), 95% Confidence Interval (CI) and *p*-value.

Explanatory variable		Estimate (days)	SE	95% CI	*P* value
Reference		31.71	6.68		
Production concept					
	Regular growers (ref)				
	Slower growers	6.50	2.57	1.25-11.60	0.014
Agricultural activities (performed during rearing cycle)					
	No (ref)				
	Yes	−2.14	2.56	−7.22-2.93	0.405
Presence of animals other than broilers on farm					
	No (ref)				
	Yes	−5.34	6.60	−18.45-7.77	0.421
Hatchery					
	A (ref)				
	B	−6.74	7.66	−21.96-8.48	0.382
	C	6.90	10.41	−13.79-27.59	0.510
	D	1.82	3.23	−4.88-8.24	0.575
	E	2.83	4.55	−6.28-11.86	0.537
	Others	0.27	4.17	−8.02-8.55	0.949
Preceding positive flock					
	No (ref)				
	Yes	−2.21	2.42	−7.01-2.60	0.365
Number of houses					
	1-3 (ref)				
	>4	−5.37	3.00	−0.60-11.35	0.078

## DISCUSSION

### Percentage of Positive Flocks

In general, the percentage of positive flocks in our study was lower as compared to national monitoring data based on random sampling of flocks (cecal samples) at slaughter in the same time period. The national *Campylobacter* monitoring reported 52% positive flocks in 2017 (in the period June-December 2017), 42% in 2018, 44% in 2019 and 34% in 2020 (data provided by NEPLUVI). Lower percentages found in our study may be caused by different factors, for example selection bias since participation in this study was on voluntary basis, or the result of (temporally) increased awareness as a result of participating in this study.

### Risk Factors Associated With Introduction and Presence of Campylobacter in Flocks

Season. Season was associated with presence of *Campylobacter* in flocks at slaughter (Table 3). This trend is also well reflected in the results of *Campylobacter* monitoring at Dutch broiler slaughterhouses (Anonymous 2022), previous national (Bouwknegt et al., 2004, Cuperus et al., 2020) and international studies (EFSA. 2020). Seasonality is also reflected in the number of cases of Campylobacteriosis in humans, both in the Netherlands (Vlaanderen et al., 2021) and internationally (EFSA/ECDC. 2021). Multiple factors might explain the seasonality of *Campylobacter* prevalence in broiler flocks (EFSA. 2020). These underlying mechanisms are still not fully understood. In a recent modelling study by Horvat et al. (2022) the seasonal effect on *Campylobacter* in poultry houses could be accurately simulated based on increased ventilation at higher temperatures. This facilitates the introduction of insects and/or dust from the neighboring environment in the broiler house, together with increased numbers of insects as a result of higher development rate in spring and summer. Ventilation as a risk factor for introduction of *Campylobacter* in poultry houses is also supported by a Dutch study on transmission of Avian influenza (Elbers et al., 2022). Additionally, poultry farmers generally enter the broiler houses more frequently in case of high temperatures, which increases the chance of Campylobacter introduction in the broiler flock. According to model 2 season did not affect the age at which chickens were first found *Campylobacter* positive.

Production concept. Slower growing breeds have in general a longer production cycle compared to regular growers. In this study the average rearing time was 52 vs. 41 d for slower vs. regular growers. Therefore, it can be expected that slower growers would be more frequently *Campylobacter* positive at slaughter age than regular growers, as a longer rearing time means more chance of exposure to *Campylobacter*. Remarkably, the opposite was observed in the univariable analysis (Table 2). Interestingly, results on the moment of introduction of *Campylobacter* in a broiler flock showed that the colonization of slower growers with *Campylobacter* was delayed by nearly a week (6.5 d, Table 5). There may be multiple factors associated with such a delay. One hypothesis could be genetic differences between regular and slower growing breeds in their susceptibility to *Campylobacter*. Differences between breeds in susceptibility for *Campylobacter* have been reported before, Li et al. (2010) found differences between 2 broiler lines (A and B) in their immune response to *C. jejuni* colonization, with one line being more resistant to *C. jejuni* colonization, however no information was provided on which breeds were tested. Hankel et al. (2018) found a lower prevalence, count and higher decrease in *Campylobacter* shedding by layer breeds as compared to broilers in an experimental study. On the other hand, a field challenge study conducted by Gormley et al. (2014) demonstrated that levels of *Campylobacter* in broiler chicken caeca were not affected by the breed. However, susceptibility or prevalence were not addressed in that study. Other hypotheses explaining the delay in *Campylobacter* colonization in slower growers could be management practices, for example, lack of thinning, lower stocking density or diet. Also, in general farmers enter the house less frequently in case of slower growers compared to regular growers. Another factor could be litter humidity, since the broiler houses with slower growing breeds have in general lower air humidity as compared to regular growing breeds. Previous studies however are inconclusive about the potential effects of litter humidity on *Campylobacter* survival (Williams et al., 2013; Robyn et al., 2015; Smith et al., 2016; Koene et al., 2019; Cuperus et al., 2020). Also in the current study many risk factors related to farm management were associated with the production concept, making it difficult to entangle the contribution of individual factors.

Preceding flock status. We found a strong association between *Campylobacter* presence in a flock at slaughter and the *Campylobacter* status of the preceding flock in the house (Table 2). In the presence of a positive flock both the broiler house and its surroundings are assumed to be heavily contaminated, especially upon poor cleaning and disinfection procedures (EFSA. 2011), leading to a higher risk of reintroduction in a consequent flock (Battersby et al., 2017, Damjanova et al., 2011). Similar genetic profiles of *Campylobacter* found in consecutive broiler flocks have been described in the literature, pointing to the scenario of spill-over between flocks instead of a new introduction (Damjanova et al., 2011). In case of spill-over between flocks one would expect that, following a positive flock, the subsequent flock may become positive at a relatively early age. However, we did not observe a difference in the age at which flocks test positive for the first time, suggesting the relevance of new introductions in the broiler house.

Mowing. The effect of mowing, increasing the risk on Campylobacter in a broiler flock at slaughter age might be explained by a potential spread of *Campylobacter* via particles from the environment (e.g. dust), or by movement of animals (mice, birds) or insects, seeking the shelter in a house or enter the house via the ventilation systems. Ventilation can be a potential introduction route for *Campylobacter* (Elbers et al., 2022). Mowing of the vegetation has been reported as a risk factor for high pathogenic avian influenza (**HPAI**) infection on laying hens farms (Garber et al., 2016) and soil disruption (e.g., tilling) in a nearby field was reported as a risk factor for HPAI in U.S. turkey farms (Wells et al., 2017).

Thinning. We did not find a strong association between thinning and *Campylobacter* presence at slaughter age or the moment of introduction of *Campylobacter*, in the univariable analyses. Due to the strong association with production concept, thinning was not included in the multivariable models. We observed that for the limited number of flocks (n=38) for which the date of thinning was known, half of the flocks (20/38) were already *Campylobacter* positive before thinning date. In other studies thinning has been reported as a major risk factor for the occurrence of *Campylobacter* infections on broiler farms (EFSA. 2020), due to breaking the biosecurity barrier (Smith et al., 2016; Georgiev et al., 2017; Millman et al., 2017). In a modelling study, discontinuation of thinning was estimated to reduce the number of contaminated broiler flocks by at least one-third (Georgiev et al., 2017). However, also age might affect the association between thinning and *Campylobacter* colonization (Russa et al., 2005). Thinned broilers are older and thus have a higher chance of becoming infected with *Campylobacter* based on their age alone.

Agricultural side activities. Our results revealed an association between presence of *Campylobacter* at slaughter age and performance of agricultural side activities by the farmer during the rearing cycle. The analysis suggested these activities as being a protective factor and decrease the risk of a positive flock. There might be underlying factors to these agricultural activities that play a role that were not included in this study, for instance having smaller farms, being more prone to rearing slower growing flocks as these require less effort from farmers, or entering chicken houses less often, because of farmers duties elsewhere.

### Multiple Introductions of Campylobacter

Additional risk factors described in the literature have been reviewed in the recent EFSA opinion (EFSA. 2020). Interestingly, various studies report different combinations, and even contradictory associations, of risk factors. Although this also depends on the selection of variables in the model, another explanation could be the occurrence of multiple introductions of *Campylobacter* into flocks. This might potentially occur, since various *Campylobacter* strains were found in one broiler flock (Damjanova et al., 2011, Vidal et al., 2016). The possibility of multiple introductions in one flock may influence the outcome and interpretation of a risk factor analysis.

### Moment of Introduction of Campylobacter

One strength of this study is in the longitudinal data on the flocks. This enabled us to determine the week during the rearing cycle when the flocks became positive, interpreted as the moment of introduction. On average, slower grower breeds became *Campylobacter* positive about a week later compared to regular growers. It should be noted that poultry farmers were asked to collect fecal samples starting in the second week of life and thus most flocks were not sampled in the first weeks. Only 54 flocks were sampled within the first 2 wk, of which 2 were positive. As flocks were sampled once a week, the exact moment (day) of introduction of *Campylobacter* in a flock could not be determined with these data. However, the results summarize well the week of age in which, on average, flocks first became *Campylobacter* positive: 5th for regular and 6th for slower growers.

### Biosecurity

Biosecurity is frequently addressed in studies on risk factors for *Campylobacter* introduction into broiler flocks. No clear associations between biosecurity measures and the presence of *Campylobacter* were found on the sampled farms (data not shown). Collection and interpretation of this information proved to be very challenging. Frequently we received limited records of activities performed on farms, for example, on the occurrence of agricultural activities. It would be worthwhile to collect insight in daily practices with the help of cameras, as applied in Canada (Racicot et al., 2011).

## CONCLUSIONS

The aim of this study was to identify introduction and presence of *Campylobacter* on Dutch broiler farms and associated risk factors, in order to advice poultry farmers how to prevent *Campylobacter* introduction. Longitudinal monitoring flocks and farms gave useful insights in the moment of introduction of *Campylobacter* in a flock and the delayed introduction in slower grower vs. regular grower flocks. The reasons behind could not be identified based on the current data. This study confirmed the importance of season as major risk factor to the presence of *Campylobacter* in a broiler flock. The relevance of mowing and preceding positive flocks as risk factors might point to the importance of ventilation in chicken houses as a potential transmission route for *Campylobacter*. It is recommended for future studies to assess contributions of these risk factors and study possible pathways more into detail and also consider multiple introductions of *Campylobacter* during a rearing cycle.

## Appendix Supplementary materials

Image, application 1

## Footnotes

Supplementary material associated with this article can be found, in the online version, at doi:10.1016/j.psj.2024.103568.
